# Evading strength-corrosion tradeoff in Mg alloys via dense ultrafine twins

**DOI:** 10.1038/s41467-021-24939-3

**Published:** 2021-07-29

**Authors:** Changjian Yan, Yunchang Xin, Xiao-Bo Chen, Daokui Xu, Paul K. Chu, Chaoqiang Liu, Bo Guan, Xiaoxu Huang, Qing Liu

**Affiliations:** 1 Key Laboratory for Light-weight Materials, Nanjing Tech University, Nanjing, China; 2 Institute of Corrosion Science and Technology, Guangdong, China; 3 School of Engineering, RMIT University, Carlton, VIC Australia; 4 Key Laboratory of Nuclear Materials and Safety Assessment, Institute of Metal Research, Chinese Academy of Sciences, Shenyang, China; 5 Department of Physics, Department of Materials Science and Engineering, and Department of Biomedical Engineering, City University of Hong Kong, Kowloon, Hong Kong China; 6 State Key Laboratory of Powder Metallurgy, Central South University, Changsha, China; 7 International Joint Laboratory for Light Alloys, College of Materials Science and Engineering, Chongqing University, Chongqing, China

**Keywords:** Mechanical properties, Metals and alloys

## Abstract

Conventional ultrafine-grains can generate high strength in Mg alloys, but significant tradeoff of corrosion resistance due to inclusion of a large number of non-equilibrium grain boundaries. Herein, an ultrafine-grain structure consisting of dense ultrafine twins is prepared, yielding a high strength up to 469 MPa and decreasing the corrosion rate by one order of magnitude. Generally, the formation of dense ultrafine twins in Mg alloys is rather difficult, but a carefully designed multi-directional compression treatment effectively stimulates twinning nucleation within twins and refines grain size down to 300 nm after 12-passes compressions. Grain-refinement by low-energy twins not only circumvents the detrimental effects of non-equilibrium grain boundaries on corrosion resistance, but also alters both the morphology and distribution of precipitates. Consequently, micro-galvanic corrosion tendency decreases, and severe localized corrosion is suppressed completely. This technique has a high commercial viability as it can be readily implemented in industrial production.

## Abstract (web summary)

Conventional ultrafine grains can generate high-strength Mg alloys, but non-equilibrium grain boundaries deteriorates their corrosion resistance. Here, the authors present ultrafine grained Mg alloys with dense twins that display high strength and reduced corrosion rate by one order of magnitude.

## Introduction

Comprising 2.7% of the earth’s crust, magnesium (Mg) with a density of approximately 23% that of steel and 66% that of aluminum is a widely used metal in the industry especially energy-efficient and environmentally friendly applications^1,2^. Mg is chemically reactive and its native passive surface layer does not offer sufficient protection against corrosion^3,4^. In addition, the relatively low mechanical strength of pure Mg cannot satisfy the demand by engineering applications. Therefore, the preparation of ultra-strong and highly corrosion-resistant Mg alloys is of great significance.

In general, corrosion resistance of Mg alloys correlates inversely with their mechanical strength, which is well demonstrated in a large number of ultrafine-grained or precipitate-hardened Mg alloys^5,6^. Mg alloys exhibit a much strong grain boundary strengthening response^7^ and an ultrafine grain structure (grain size < 1 µm) is frequently employed to obtain ultra-strong Mg alloys. Severe plastic deformation (SPD) is an effective method to produce ultrafine grains. Although the poor working ability of Mg alloys hampers the applicability of many SPD techniques, there has been a success in preparing ultrafine-grained Mg products using high-pressure torsion (HPT) and surface mechanical attrition treatments (SMAT)^5,8^. Nevertheless, these ultrafine grains consist of a large number of non-equilibrium grain boundaries which produce detrimental effects on corrosion resistance. For example, corrosion rates of pure Mg and Mg-1Ca alloy increase by more than one order of magnitude after SMAT^5^. Furthermore, these ultrafine-grained Mg alloys prepared by SPD are often too small for industrial applications and it remains a great challenge to fabricate ultrafine-grained Mg alloys with high corrosion resistance using techniques suitable for mass production. In addition, the secondary phases in precipitate-hardened Mg alloys often serve as cathodes that can generate profound galvanic corrosion, and in particular, an inhomogeneous distribution of precipitates often leads to severe localized corrosion and rapid loss of mechanical integrity^9^.

Deformation twins are also used to refine grains and strengthen Mg alloys^10^. Nevertheless, the production of a high density of ultrafine twins in Mg alloys is rather difficult and the strengthening effect by twins is quite limited as well because the high mobility of twin boundary under strain often leads to twin thickening and coalesce of the same twin variant^10^.

In this work, a strategy to prepare ultrafine-grained Mg AZ80 (grain size ~300 nm) consisting of dense ultrafine $\{10\bar{1}2\}$ twins via a carefully designed multi-directional compression is described and demonstrated. Compared to non-equilibrium grain boundaries, the low energy of twin boundaries effectively circumvents the detrimental effects of non-equilibrium grain boundaries on corrosion resistance. In addition to a high strength of up to 469 MPa, such an ultrafine twin (UFT) structure decreases corrosion rate by one order of magnitude and inhibits severe localized corrosion completely. The proposed mass-production feasible process enables the production of Mg alloys with both high strength and high corrosion resistance.

## Results

### Ultrafine twin structure

**Fig. 1:**
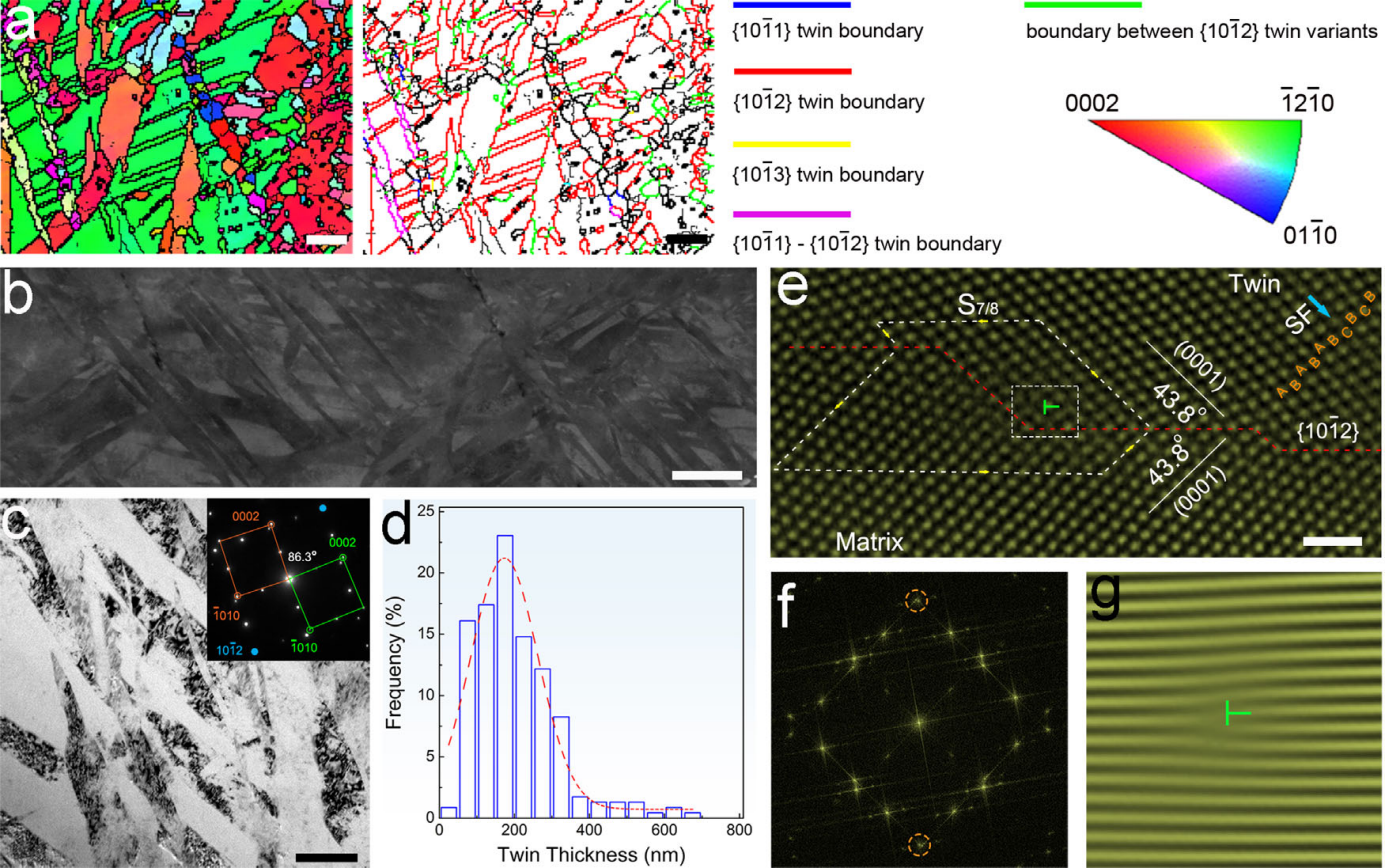
Microstructure of high-density twins. **a** Inverse pole figure map and boundary misorientation map for UFT-4, scale bar = 2 μm. **b** Electron channeling contrast image of twins in UFT-4, scale bar = 2 μm. **c** Transmission electron microscopy (TEM) image, scale bar = 500 nm. **d** Distribution of twin thickness in UFT-4; (**e**) HAADF-STEM image for $\{10\bar{1}2\}$ twin boundary, scale bar = 1 nm; (**f**) Fast Fourier transform pattern; (**g**) Dislocation displayed by the IFFT lattice fringe image generated by mask $\{10\bar{1}2\}$ diffractions of two crystals in an image (**f**). Solid solution-treated AZ80 with compression passes for 1, 3, 6, and 12 are designated as UFT-1, UFT-2, UFT-3, and UFT-4, respectively. SF and *S*_7/8_ in e denote stacking fault and twinning dislocation around the step, respectively.

$\{10\bar{1}2\}$ twinning can be the predominant deformation mode under certain loading conditions of highly-textured Mg alloys. Nevertheless, the high mobility of twin boundaries under stress often generates a small density of thick twin lamellae^10^. Although the twins can be employed to refine the grains in Mg alloys, obtaining a dense UFT structure is still a big challenge. The average grain size in the pristine materials is approximately 33 μm. An ultrafine grain structure (average grain size ~300 nm) consisting of high density ultrafine $\{10\bar{1}2\}$ twins (average lamellar spacing ~200 nm) is observed from UFT-4 after being subjected to 12 passes of multi-directional compression at room temperature (Fig. 1a–d and Supplementary Fig. 1k). The density and size of twins depend on the number of compression passes (Supplementary Fig. 1a–j). Finer and more twins are observed after more compression passes and a twin spacing of ~2.6 µm and average grain size of ~8.8 µm are observed after 1 pass. After 6 passes, a twin spacing of ~560 nm and average grain size of ~760 nm are observed. There is an obvious texture in UFT samples, while the maximum intensity of texture components is largely weakened, compared to the starting material (Supplementary Fig. 2). The twin bands either are parallel to or intersect with each other (Fig. 1c). The result of transmission Kikuchi diffraction-electron back-scattered diffraction (TKD-EBSD) shows that the twins are mainly $\{10\bar{1}2\}$ $< 10\bar{1}1 >$ twins (Fig. 1a). The fine structure is further analyzed by atomic-resolution high-angle annular dark-field scanning transmission electron microscopy (HAADF-TEM) (Fig. 1e–g). The $\{10\bar{1}2\}$ twin boundary contains an interfacial step in the $< 1\bar{2}10 >$ projection and a misfit dislocation associated with the interfacial step can be identified from the inverse fast-Fourier transform (IFFT) image. According to the step heights, the twinning dislocations around the steps are denoted as *S*_7/8_, where 7 and 8 are the number of the twinning planes on the matrix side and twin crystal side, respectively. A stacking fault indicated by the blue arrow is observed from the twin interior.

**Fig. 2:**
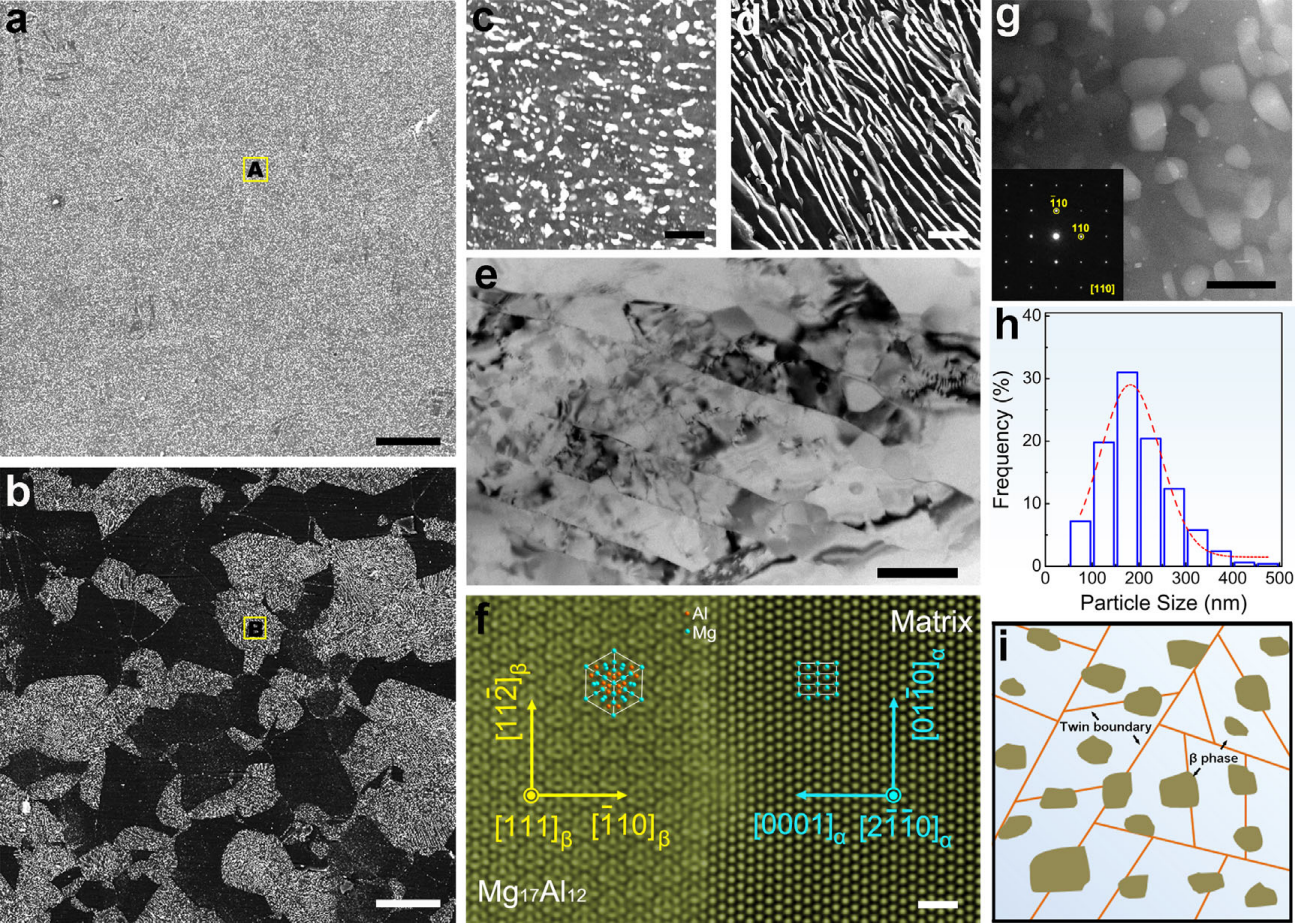
Microstructure after aging at 180 °C for 24 h. Scanning electron microscopy (SEM) images of (**a**) Aged UFT-4 and (**b**) AZ80-T6, scale bar = 20 μm in (**a**) and (**b**); (**c**) and (**d**) High-magnification SEM images of regions A and B, scale bar = 2 μm in (**c**) and (**d**); (**e**) TEM image of aged UFT-4, scale bar = 500 nm; (**f**) atomic resolution HAADF-STEM and (**g**) HAADF-STEM images of the precipitates in aged UFT-4, scale bar = 1 nm in **f** and scale bar = 500 nm in (**g**); (**h**) Size distribution of the precipitates in aged UFT-4. **i** Schematic diagram showing the microstructure of aged UFT-4.

In the T6 treated AZ80 (AZ80-T6), an inhomogeneous distribution of β-Mg_17_Al_12_ composed of plate-like discontinuous precipitates in the light areas and needle-like continuous precipitates in the dark regions (Fig. 2b and Supplementary Fig. 3a, b, e, and 4b) can be discerned. The discontinuous precipitates have a length of several to tens of micrometers and most of the continuous precipitates are ~200 nm long. In contrast, there is a homogeneous distribution of β-Mg_17_Al_12_ particles with an average diameter of ~200 nm in the aged UFT-4 (Fig. 2a, g and h, and Supplementary Fig. 4a and 5). The TEM image in Fig. 2e indicates that the particles appear from both the twin boundaries and the twin interior. The distribution and homogeneity of the precipitates are related to the density of twin boundaries. The precipitates exhibit a very homogeneous distribution after 6 or 12 compression passes (Supplementary Fig. 6). Although thermomechanical processing alters the morphology and distribution of precipitates, it does not vary the crystalline structure and orientation relationship with the α-Mg matrix (Fig. 2f and Supplementary Fig. 3d and g). Under the testing conditions, the Burgers orientation relationship of β-Mg_17_Al_12_ and α-Mg is ${(\bar{1}10)}_{\beta }$ // (0001)_α_, [111]_β_ // ${[2\bar{1}\bar{1}0]}_{\alpha }$.

### High corrosion resistance

**Fig. 3:**
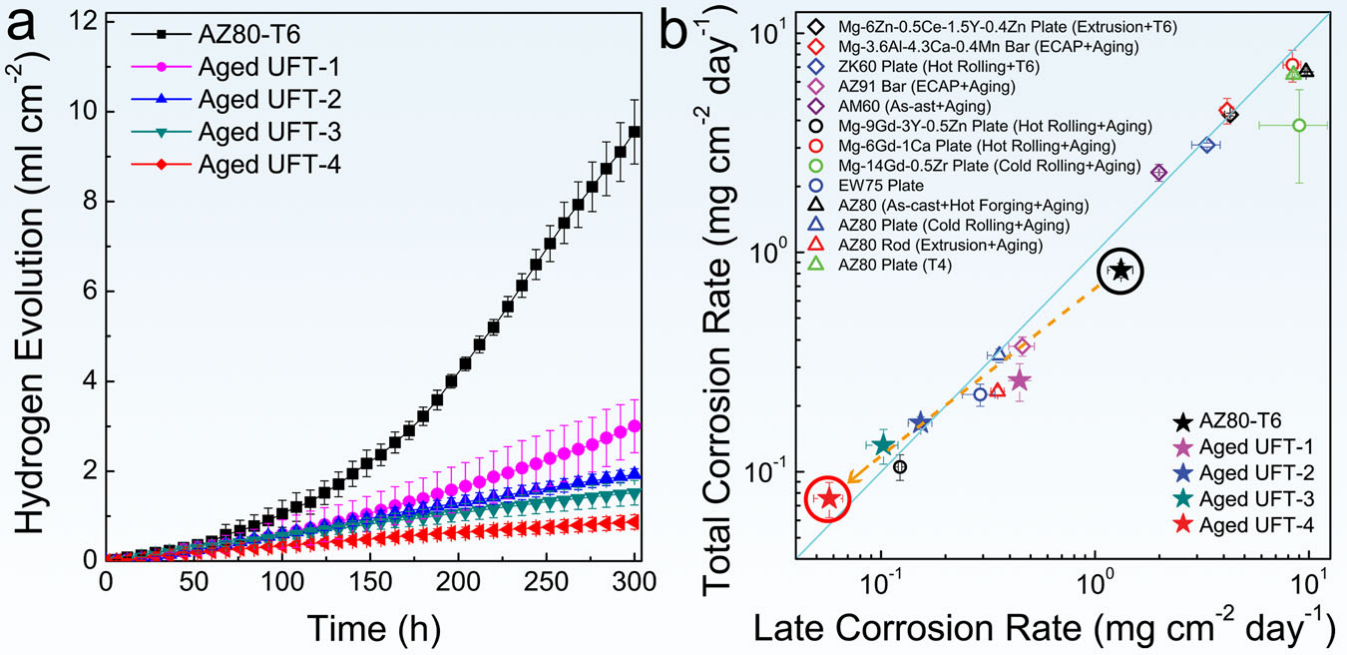
Corrosion rates in 3 wt.% NaCl solution. (**a**) Corrosion rates as a function of time determined from the volume of hydrogen emitted. **b** Comparison of the corrosion rates between the aged UFT samples and typical precipitate-hardened Mg–Al, Mg–Zn, and Mg–RE alloys. Error bars in (**a**) and (**b**) represent one standard deviation of the data from the mean. The x-axis is the corrosion rate for the last 24 h and the y-axis is the average corrosion rate for 300 h. If the symbol is above the blue line, the corrosion rate decreases with time, otherwise it increases with time. ECAP in (**b**) refers to an equal channel angular process.

**Fig. 4:**
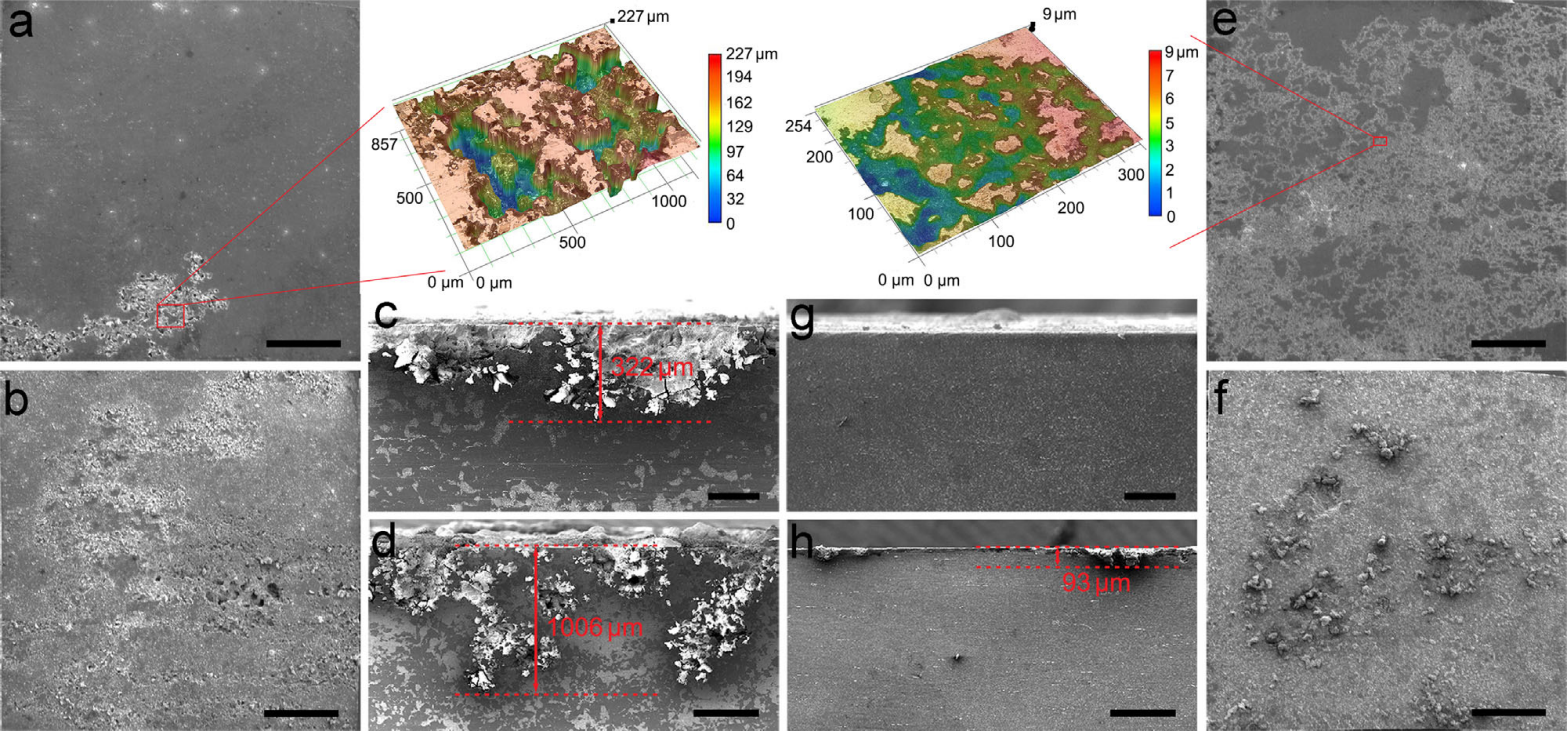
Corrosion morphology after exposure to 3 wt.% NaCl solution. AZ80-T6 after (**a**) 24 h and (**b**) 168 h, scale bar = 2 mm in **a** and **b**; aged UFT-4 after (**e**) 24 h and (**f**) 168 h, scale bar = 2 mm in (**e**) and (**f**). Cross-sectional views of AZ80-T6 after (**c**) 24 h and (**d**) 168 h, scale bar = 200 μm in (**c**) and scale bar = 500 μm in (**d**). Cross-sectional views of aged UFT-4 after (**g**) 24 h and (**h**) 168 h, scale bar = 200 μm in (**g**) and scale bar = 500 μm in (**h**).

AZ80-T6 shows a bigger corrosion rate than the aged UFT samples (Fig. 3a). In particular, the average corrosion rate of aged UFT-4 (0.0755 mg cm^−2^ day^−1^) after 300 h is one order of magnitude less than that of the AZ80-T6 (0.8289 mg cm^−2^ day^−1^). A comparison of the precipitate-hardened Mg–Al, Mg–Zn, and Mg–RE alloys after different thermomechanical treatments shows that the aged UFT-4 has the lowest corrosion rate (Fig. 3b). The corrosion rate of AZ80-T6 increases gradually with immersion time, but not so for the aged UFT-3 and UFT-4. AZ80-T6 suffers from intense localized corrosion manifested by many deep corrosion pits (Fig. 4a and b). The cross-sectional views disclose that the localized corrosion pits propagate by undermining the precipitates and the depth of corrosion pits exceeds 300 µm after immersion for 24 h (Fig. 4c) and 1000 µm after 168 h (Fig. 4d). In contrast, homogeneous and shallow corrosion is observed from aged UFT-4 (Fig. 4g and h).

### High strength and plasticity

**Fig. 5:**
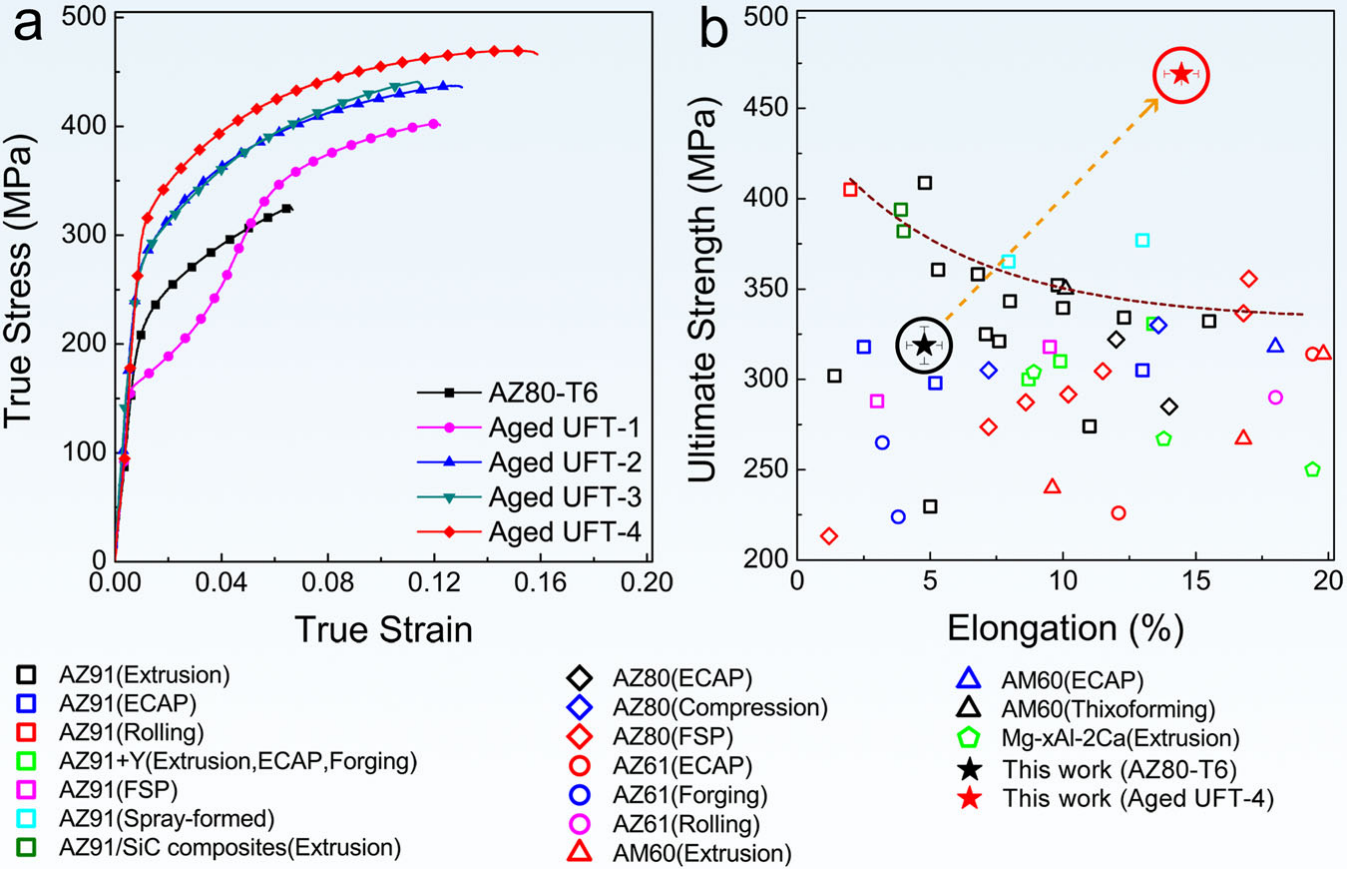
Tensile mechanical properties at room temperature. **a** Stress-strain curves under tension along the transverse direction (TD). **b** Comparison of the ultimate tensile strengths and uniform elongations between the aged UFT-4 and typical precipitate-hardened Mg–Al alloys subjected to different thermomechanical processes (AZ91 after Extrusion^27,28^, ECAP^27^, Rolling^29^, FSP^30^, Spray formed^31^. AZ91+Y after Extrusion^32^, ECAP^33^, Forging;^33^ AZ91/SiC composites after Extrusion;^34^ AZ80 after ECAP^35^, Compression^36^, FSP;^37,38^ AZ61 after ECAP^39^, Forging^40^, Rolling;^41^ AM60 after Extrusion^42,43^, ECAP^44^, Thixoforming^45^. Mg-xAl-2Ca after Extrusion^46^). Error bars in a and b represent one standard deviation of the data from the mean. ECAP refers to equal channel angular process and FSP denotes friction stir processing.

The UFT samples possess larger tensile strength as well as better plasticity as shown in Fig. 5a and b. The ultimate tensile strength of aged UFT-4 is up to 469 MPa that reaches that of many Mg alloys containing a high content of rare-earth (RE) elements. The ultimate tensile strength of most high-strength Mg–RE alloys with > 10% RE is 400–500 MPa. Although the strength of some Mg–RE alloys approaches 500 MPa, there is often an elongation below 5%^11^. A comparison of the ultimate tensile strength between the aged UFT-4 and typical Mg–Al precipitate-hardened alloys after different thermomechanical processes shows that the aged UFT-4 has the highest ultimate tensile strength with similar contents besides desirable elongation. With regard to the highly-textured Mg products, there is often a strong tension-compression yield asymmetry, a big difference between the tensile yield strength and compressive one, which greatly affects the applications of Mg products. A lower tension-compression yield asymmetry measured as the ratio of the compressive yield strength (CYS) to tensile yield strength (TYS) (Supplementary Fig. 7 and Supplementary Table 1) is observed from aged UFT-4. CYS/TYS is ~0.8 for the aged UFT-4 and is closer to 1 than that of AZ80-T6 (approximately 0.58), indicating a much lower mechanical anisotropy.

## Discussion

### Origin of high corrosion resistance

Localized corrosion damage is one of the major problems affecting material strength and may produce difficulty in predicting catastrophic failure of structures^12^. Precipitate-hardened Mg alloys are vulnerable to galvanic corrosion between precipitate and α-Mg matrix giving rise to a high tendency of heavy localized corrosion. Mg–Al alloy series, including AZ80 and AZ91, contains an appreciable amount of β-Mg_17_Al_12_ which is approximately 0.45 V nobler than that of α-Mg matrix and serves as cathodic part^9^. It is well known that β-Mg_17_Al_12_ plays two contradictory roles in the corrosion responses of Mg–Al alloys^9^. It can serve as a cathode in micro-galvanic actions leading to accelerated corrosion of the surrounding α-Mg matrix (anode). On the other hand, it can be a barrier impeding corrosion propagation in the case of a net-like distribution. The knowledge has been built from studies of the as-cast alloys. The morphology and distribution of β-Mg_17_Al_12_ after the T6 treatment in the present study are different from those of the as-cast sample (Supplementary Fig. 3). Zhao et al.^6^ considered that the lamellar discontinuous precipitates after T6 treatment of Mg AZ91 can act as a barrier to hinder corrosion propagation in the matrix and reduce corrosion rate. However, no direct evidence has been provided.

**Fig. 6:**
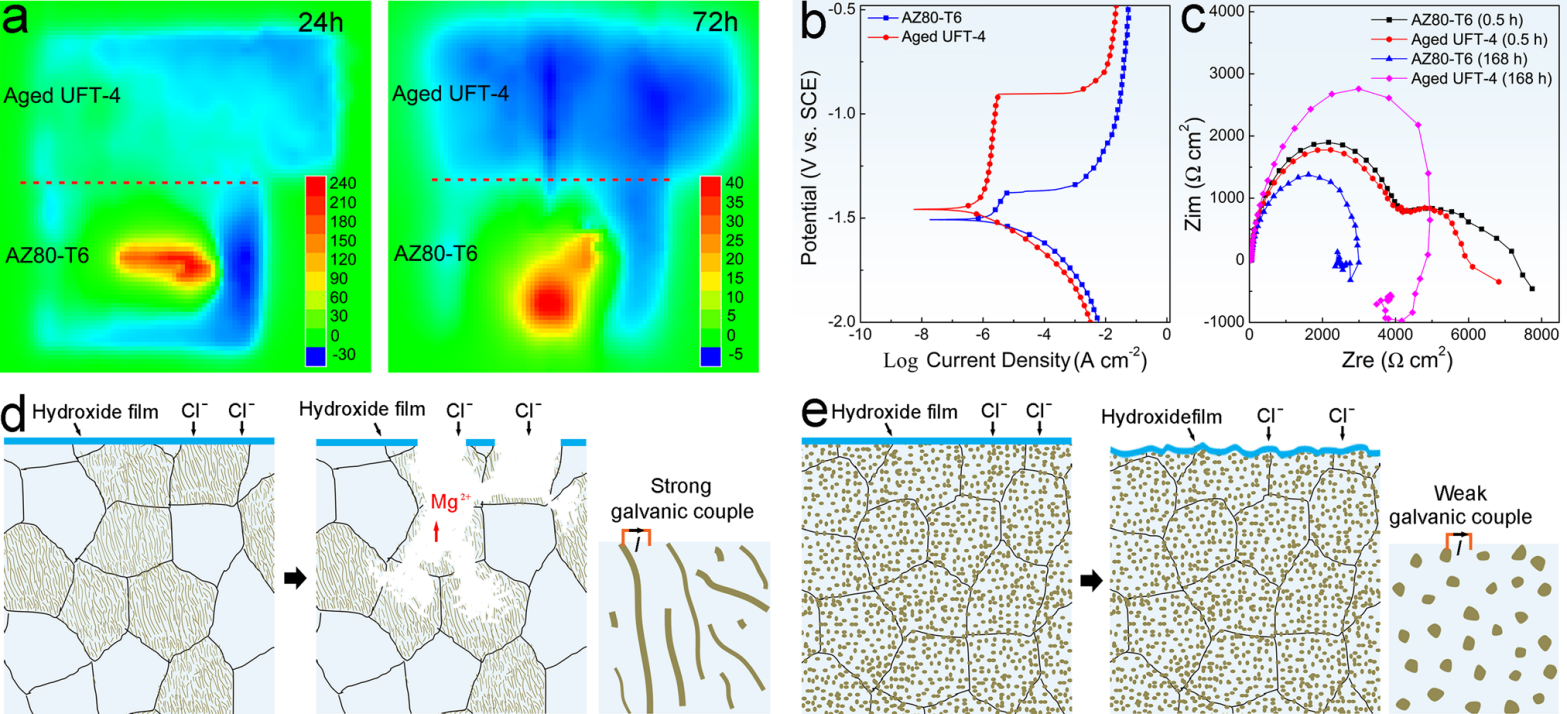
Electrochemical corrosion characteristics and corrosion mechanism. **a** SVET measurement of the coupled AZ80-T6 and aged UFT-4 after immersion for 24 h and 72 h. **b** Potential dynamic polarization curves and (**c**) EIS spectra of AZ80-T6 and aged UFT-4 after immersion for 0.5 h and 168 h; Schematic illustration of the corrosion mechanism: (**d**) AZ80-T6 and (**e**) aged UFT-4. SCE in (**b**) represents saturated calomel electrode.

Here, *in-situ* observation of the corrosion evolution in AZ80-T6 (Supplementary Fig. 8) shows that corrosion occurs preferentially in regions with discontinuous β-Mg_17_Al_12_ leading to a strong localized corrosion and deep pits. SEM observation afterward confirms that corrosion concentrates at the precipitates and propagates bypassing the regions with discontinuous precipitates and undermining of precipitates forms deep localized corrosion pits (Supplementary Fig. 9a–b, 10a–b, and 11a–b). In contrast, this type of heavy localized corrosion is absent from the aged UFT-4 (Supplementary Fig. 9c–d, 10c–d, and 11c–d). In general, micro-galvanic acceleration depends on the area ratio between the cathode (β) and anode (α) and a larger ratio produces more severe micro-galvanic corrosion. That is, finer particles will lead to a lower tendency to micro-galvanic corrosion^13^. Nevertheless, the critical size for β-Mg_17_Al_12_, at which localized corrosion can be reduced markedly, has not been reported yet. In addition, a homogeneous distribution of β-Mg_17_Al_12_ has proven to be important to reduce galvanic corrosion^14^. As shown in Fig. 2, a great number of fine precipitates (approximately 200 nm) exist along with a very uniform distribution in aged UFT-4. In contrast, discontinuous β-Mg_17_Al_12_ is substantially coarse (approximately several to tens of micrometers) in AZ80-T6, along with a quite inhomogeneous distribution. Evidently, it will generate weak micro-galvanic couples in aged UFT-4 but strong counterparts in AZ80-T6 at localized regions. For confirmation, a scanning vibrating electrode technique (SVET) is performed on the coupled AZ80-T6 and aged UFT-4 and the results show that AZ80-T6 acts as an anode for the anodic dissolution reaction (Fig. 6a). The optical observation accompanied by SVET measurement further corroborates that AZ80-T6 suffers from severe corrosion, but aged UFT-4 hardly corrodes (Supplementary Fig. 12). The SVET results indicate that there is stronger micro-galvanic corrosion between β-Mg_17_Al_12_ and α-Mg matrix in AZ80-T6 compared to aged UFT-4.

Potentiodynamic polarization curves in Fig. 6b show a strong passivation in aged UFT-4 but not AZ80-T6. That is, a protective layer is more easily formed on aged UFT-4 during immersion in the electrolyte. Similar trends are observed from the results obtained by electronchemical impedance spectroscopy (EIS) and larger values of R_t_ of aged UFT-4 compared to AZ80-T6 are observed (Supplementary Fig. 13 and Supplementary Tables 2 and 3). This also reveals a better protective layer on aged UFT-4. X-ray photoelectron spectroscopy (XPS) indicates that the corrosion layer contains Al and Mg hydroxide or oxide in both aged UFT-4 and AZ80-T6 after exposure to 3 wt.% NaCl (Supplementary Fig. 14). Nevertheless, EDS maps (Supplementary Fig. 10–11) further indicate that the Al oxide and/or hydroxide accounts for a higher fraction in the corrosion product layer. A uniform distribution of fine β-Mg_17_Al_12_ mitigates the micro-galvanic corrosion reaction and contributes to the formation of a complete and effective protective layer after corrosion. The absence of localized corrosion is an important reason why corrosion rates of aged UFT samples are smaller.

Grain refinement is considered to reduce the corrosion rate of Mg alloys. The mechanism involves three aspects^15^. Firstly, grain refinement accelerates the formation of the surface bilayer of magnesium oxide and hydroxide to reduce the corrosion rate. Secondly, a large fraction of grain boundaries relieves the stress between the corrosion layer and matrix consequently decreasing the cracking tendency of the protective layer. Thirdly, the impurities and solutes tend to segregate at grain boundaries thus contributing to higher corrosion resistance. In fact, grain refinement does not always decrease the corrosion rate of Mg alloys. For example, although an Mg AZ31 after SMAT contains nano-scaled grains in the surface layer, the corrosion rate increases by a factor of 2^16^. The cold SPD methods such as SMAT and HPT are effective ways to produce ultrafine grains, while generating a high density of dislocations and non-equilibrium grain boundaries which bring about substantial grain reduction to the submicron and even nanoscale levels but have a much higher energy than the equilibrium ones^17^. Due to the poor corrosion resistance of Mg alloys, non-equilibrium grain boundaries often lead to a severe anode dissolution in SPDed Mg alloys.

Twin boundary is an effective structure to refine grains and denser twins contribute to a higher strength. Compared to the non-equilibrium grain boundaries generated by SPD, there is a much lower energy for twin boundaries. As shown in Supplementary Fig. 15, a dislocation density of 1.9 × 10^13^ m^−2^ is observed for UFT-4, evidently lower than those for Mg alloys which are subjected to typical SPD processes or conventional extrusion. Therefore, ultrafine grains consisting of high-density twins are suspected to achieve ultra-strong and corrosion-resistant Mg alloy. With regard to *hcp*-structured Mg alloys, $\{10\bar{1}2\}$ twinning can be the predominant deformation mode that facilitates the preparation of twin structures. However, the preparation of dense ultrafine twins is rather difficult, as the $\{10\bar{1}2\}$ twin boundary has a high mobility under strain. Twin thickening and coalesce of the same twin variant will generate thick and low-density twin lamellae^10^. Varying the strain path of reloading can lead to secondary twinning in the primary twins. For example, compression along the TD generates $\{10\bar{1}2\}$ primary twins and re-compression along the rolling direction (RD) forms many $\{10\bar{1}2\}$-$\{10\bar{1}2\}$ twins within the $\{10\bar{1}2\}$ primary twins. The formation of secondary twins further subdivides the twins and generates denser and finer twins. Due to the hierarchical twin structure, the strain-path changed reloading is also accompanied by detwinning, the shrinking of twin lamellae^10^. A straining mode characterized by recycling of low strain (3%) and then high strain (6.5%) is carefully designed, which is found to be very effective to suppress detwinning. As a result, dense ultrafine twins are successfully prepared by a multi-directional compression process described here. As shown in Supplementary Fig. 1, the spacing of the twin lamellae decreases gradually and the density of twins increases after more compression passes. That is to say, in order to generate dense and ultrafine $\{10\bar{1}2\}$ twins, it is important to design the proper compression paths and strain levels.

Similar to grain refinement by equilibrium grain boundaries, grain refinement by twin boundaries can improve corrosion resistance. According to the study on precipitate-free Mg alloys, grain refinement cannot dramatically reduce the corrosion rate. For example, the corrosion rate of a fine-grained Mg AZ31 (grain size ~600 nm) is about half of that of a coarse-grained one (grain size ~20 μm)^18^. Therefore, the main mechanism for the low corrosion rate observed from aged UFT samples is ascribed to the suppression of localized corrosion by the uniform distribution of fine β-Mg_17_Al_12_. Hence, the UFT structure is an ideal ultrafine grain structure with high corrosion resistance for precipitate-hardened Mg–Al alloys.

It is worth mentioning that ultrafine-grained Mg alloys prepared through conventional SPD techniques such as HPT, SMAT, and ECAP are often too small for industrial applications. However, multi-directional compression allows the preparation of meter-scaled products using an industrial forging machine. In general, a strong basal texture is essential for the preparation of high-density twins owing to the pole nature of deformation twinning. Basal texture can be easily formed in hot-rolled plates, extruded bars, and hot-forged blocks of Mg alloys. To satisfy staining on three axes, forged blocks are ideal raw materials when multi-directional compression is implanted in industrial production. Standard rolling, extrusion, and forging are generally performed at elevated temperatures, which hinders the formation of ultrafine grains. Given the poor cold working capability of Mg alloys, many cold working processes cannot be applied for the preparation of ultrafine grains. Nevertheless, a careful design of strain path for multi-directional compression can effectively activate twinning nucleation, generating an ultrafine grain structure with a low plastic strain. For example, an overall strain of 57% in UFT-4 refines the grain size down to 300 nm. Previously, ultra-strong Mg alloys are often prepared by an addition of a high content of expensive RE. The multi-directional compression is an easy-to-operate cold working process and hence is cost-efficient.

### Origin of high strength

The strength of precipitate-hardened Mg alloys originates from strengthening by the precipitate, solid solution, and grain boundaries. The strengthening by precipitates, Δ*σ*_or_, can be calculated using the Orowan model:^19^

1 $$\varDelta {\sigma }_{{{{{{\rm{or}}}}}}}=\frac{MGb}{2\pi \sqrt{1-v}\left(\frac{0.779}{\sqrt{f}}-0.785\right){d}_{{{{{{\rm{t}}}}}}}}\,{{{{\mathrm{ln}}}}}\,\frac{0.785{d}_{{{{{{\rm{t}}}}}}}}{b}$$

where *M* is the Taylor orientation factor, *G* is the shear modulus of the magnesium matrix phase, *b* is the magnitude of the Burgers vector of dislocations, *v* is Poisson’s ratio, *f* is the volume fraction of precipitates, and *d*_t_ is the uniform diameter of spherical particles. Concerning solid solution strengthening, the increment Δ*σ*_SS_ for yield strength can be estimated by the following formula:^20^

2 $$\varDelta {\sigma }_{{{{{{\rm{SS}}}}}}}=M\sum {m}_{x}{c}_{{{{{{\rm{x}}}}}}}^{1/2}$$

where *m*_x_ is the potency factor for solute *x*, and *c*_x_ is the atomic concentration of solute *x*. Generally, apparent hardening by grain boundaries, Δ*σ*_HP_, is expressed by the classical Hall–Petch relationship:^7^

3 $$\varDelta {\sigma }_{{{{{{\rm{HP}}}}}}}=k{d}^{-1/2}$$

where *d* is the grain diameter and *k* is the Hall–Petch slope. The Orowan model describes the hardening effect against slips by a uniform distribution of precipitates. Considering that there is not a uniform distribution of precipitates in aged UFT-1 and UFT-2, only the contributions of different strengthening mechanisms for aged UFT-3 and UFT-4 are calculated. The value of *M* and stress for dislocation gliding within the grain interior used in the present study is from Ref. ^20^. Orowan strengthening is about 93 MPa and solid solution strengthening is ~14 MPa (the small amount of Zn in the matrix is neglected). Grain boundary strengthening in aged UFT-3 and UFT-4 provides the most significant strengthening, approximately 164 MPa for aged UFT-3 and 199 MPa for aged UFT-4. Compared to *fcc*-structured and *bcc*-structured metals, there is a more pronounced grain boundary strengthening in *hcp*-structured materials. For example, the Hall–Petch slope of pure Al is 40–60 MPa μm^1/2^, whereas that of pure Mg is 120–300 MPa μm^1/2^ ^21^. That is, grain boundary strengthening plays a more important role in Mg alloys. Therefore, a high density of ultrafine twins is crucial to achieve ultra-strong Mg alloys.

The high ultimate tensile strength in UFT samples mainly comes from a high yield strength and a strong strain hardening response. The strain hardening responses in aged UFT-3 and UFT-4 are over 150 MPa. For many ultrafine-grained materials, strain hardening after yielding is low and even nonexistent. The main reason is the high dislocation density in the ultrafine grains leading to a low dislocation storage efficiency inside the grains during reloading, especially in the presence of dynamic recovery^22^. An important disadvantage of ultrafine-grained metals is the relatively low plasticity. For example, the elongation of the AZ61 alloy (grain size of 1.4 μm) after high-ratio differential speed rolling is only 3%^23^. The low plasticity mainly stems from a high density of dislocations after SPD and as a result of accumulation and interactions of pre-existing dislocations, obstacles are created to render propagation of dislocations difficult. Consequently, localized shear banding is induced to accommodate plastic deformation resulting in plastic instability and onset of necking. In contrast, the aged UFT-4 has a high plasticity that is even much better than that of AZ80-T6. The UFT structure has a smaller dislocation density (Supplementary Fig. 15) and possesses a high capacity of dislocation storage within grains. Molecular dynamic simulations have suggested that dislocations approaching a coherent or semi-coherent twin boundary can transfer into the adjacent twin by a cross-slip at the boundary^24,25^, which facilitates the storage of these dislocations, thereby accommodating considerable strain hardening. Simultaneously, impediments to the easy flow of dislocations at twin boundaries and a continual loss of coherency benefit markedly to the increase of strength and ductility^26^. The microstructure of aged UFT-4 subjected to a 3% tensile strain was examined by TEM (Supplementary Fig. 16). A traversing of dislocation across twin boundaries is observed, which contributes to a better ductility^26^.

We have successfully solved the challenging issue of fabricating a high density of ultrafine twins in Mg AZ80 using a mass-production process. This ultrafine twin structure effectively evades the strength-corrosion tradeoff in ultrafine-grained Mg alloys. This method is also potential to be applied to other Mg alloys. Our results demonstrate that the preparation of ultra-strong and highly corrosion-resistant Mg alloys for industrial applications is now possible.

## Methods

### Sample preparation

A hot-rolled Mg AZ80 plate (Supplementary Fig. 17a and b) with a thickness of 40 mm was cut into blocks of 40 mm (RD) × 40 mm (TD) × 40 mm (ND, normal direction), solid-solution-treated at 400 °C for 24 h, and quenched in cold water. The solution-treated sample was subjected to multi-directional compressions with 1 pass, 3 passes, 6 passes, and 12 passes as schematically illustrated in Supplementary Fig. 17c. The strainning mode characterized by recycling of low strain (3%) and then high strain (6.5%) was adopted and found to be more effective to generate finer twin lamellae. Both the solid-solution-treated samples and pre-deformed samples were aged at 180 °C for about 24 h to obtain the peak-aged state. The age hardening response as a function of time was assessed on the Vickers hardness teater (EVERONE, NH-5L).

### Mechanical tests

The tension and compression tests along TD were carried out at room temperature on the Shimadzu AG-X machine at a strain rate of 0.001 s^−1^. Specimens for compression tests were blocks of 9 mm (RD) × 9 mm (ND) × 12 mm (TD) and those for tension tests had a dog bone shape with a gauge length of 13 mm and cross-sectional dimensions of 4 mm × 2.5 mm. Each mechanical test was repeated three times.

### Corrosion tests

Dissolution of Mg emits hydrogen and a hydrogen evolution method was implemented to measure the corrosion rate as schematically illustrated in Supplementary Fig. 18. For each test, four blocks of 10 mm × 10 mm × 2 mm were soaked in 500 ml of the electrolyte containing 3 wt.% NaCl for 300 h at ambient temperature and the volume of emitted hydrogen was monitored as a function of time. Each test was repeated more than two times. For comparison, the corrosion rates of other 13 Mg alloys (Mg-6Zn-0.5Ce-1.5Y-0.4Zn plate after extrusion and T6 treatment, Mg-3.6Al-4.3Ca-0.4Mn bar after ECAP and aging treatment, ZK60 plate after hot-rolling and T6 treatment, AZ91 bar after ECAP and aging treatment, AM60 ingot after aging treatment, Mg-9Gd-3Y-0.5Zn plate after hot-rolling and T6 treatment, Mg-6Gd-1Ca plate after hot-rolling and T6 treatment, Mg-14Gd-0.5Zr plate after cold-rolling and T6 treatment, EW75 plate, AZ80 after hot-forging and T6 treatment, AZ80 plate after cold-rolling and T6 treatment, AZ80 rod after extrusion and aging, AZ80 plate after T4 treatment) were also tested at the same condition. The electrochemical corrosion characteristics in 3 wt.% NaCl were evaluated by potentiodynamic polarization tests, EIS, and SVET. Potentiodynamic polarization tests were conducted after exposure to the electrolyte for 0.5 h on a Gamry Reference 600+ using a scanning rate of 1 mV s^−1^. EIS test was conducted after immersion for 0.5 h and 168 h with a frequency range from 100 kHz to 10 mHz. For SVET measurement (Applicable Electronics Inc., USA), AZ80-T6 and aged UFT-4 samples were sealed together using epoxy resin and polished.

### Microstructure examination

Thin specimens for TEM and HAADF-TEM observation were prepared by mechanical polishing from 500 μm to 60–80 μm, punching into 3 mm diameter discs, and ion-beam milling using Gatan 695. TEM was carried out on FEI Tecnai G^2^ F20. HAADF-STEM and EDS were performed on FEI Titan G^2^ 60–300 ChemiSTEM. Microstructure and crystallographic orientations were analyzed by TKD-EBSD on SEM (JEOL JSM-7800) equipped with an HKL-EBSD system using a step size of 0.09 μm. The sample preparation process for TKD-EBSD mapping was similar to that for TEM. Electron channeling contrast image and EDS maps were recorded on Zeiss Auriga SEM-FIB. The surface suitable for electron channeling contrast imaging was sputter-cleaned with an argon gun Gatan 697. Because precipitates are invisible on the surface of corroded regions, samples were re-etched in an acetic picral solution (2 ml acetic acid + 1 g picric acid + 2 ml of H_2_O + 16 ml of ethanol) to show the second phase. *In-situ* corrosion process and corrosion morphology were observed under a stereo optical microscope (Keyence VHX 2000) to obtain 2D and 3D images. The chemical states of corrosion products were determined by XPS (ESCALAB250Xi, Thermo Fishes Scientific, USA) using Al K_α_ X-ray corresponding to 1486.71 eV photons.

## Supplementary information

Supplementary Information

## Data Availability

All relevant data supporting the findings of this study are contained in the paper and its Supplementary Information files. All other relevant data are available from the corresponding author on request.

## Code availability

All codes are included in the paper.
