# Supplementary material for: Evading strength-corrosion tradeoff in Mg alloys via dense ultrafine twins
Source: Nat Commun. 2021 Jul 29;12:4616. doi: 10.1038/s41467-021-24939-3 (PMC8322409; doi:10.1038/s41467-021-24939-3)
Supplement: Supplementary file 1 — Supplementary Information [file 41467_2021_24939_MOESM1_ESM.pdf]

Supplementary Information

Evading strength-corrosion tradeoff in Mg alloys via dense ultrafine twins

Changjian Yan [1, 2], Yunchang Xin [1*], Xiao-Bo Chen [3], Daokui Xu [4*], Paul K. Chu [5], Chaoqiang Liu [6], Bo Guan [7], Xiaoxu Huang [7], Qing Liu [1]

[1] Key Laboratory for Light-weight Materials, Nanjing Tech University, Nanjing 210009, China

[2] Institute of Corrosion Science and Technology, Guangdong 510070, China

[3] School of Engineering, RMIT University, Carlton 3053, VIC, Australia

[4] Key Laboratory of Nuclear Materials and Safety Assessment, Institute of Metal Research, Chinese Academy of Sciences, Shenyang 110016, China

[5] Department of Physics, Department of Materials Science and Engineering, and Department of Biomedical Engineering, City University of Hong Kong, Tat Chee Avenue, Kowloon, Hong Kong, China

[6] State Key Laboratory of Powder Metallurgy, Central South University, Changsha 410083, China

[7] International Joint Laboratory for Light Alloys, College of Materials Science and Engineering, Chongqing University, Chongqing 400030, China

[*] Corresponding authors: Yunchang Xin (ycxin@cqu.edu.cn); Daokui Xu (dkxu@imr.ac.cn);

Supplementary Notes

Generally, in solid-solution treated AZ series, continuous precipitation is favored at high aging temperature above 350 ºC and discontinuous precipitation is prevalent at low aging temperature below 150 °C, whereas continuous and discontinuous precipitation dominates the microstructure at intermediate temperature[1]. Colonies of discontinuous lamellar precipitates show anisotropy growth from the grain boundaries and continuous needle-like precipitates always precipitate inside the grains. In the present study, the aging temperature 180 °C is a typical intermediate temperature and both continuous and discontinuous precipitates are observed from AZ80-T6 (Supplementary Fig. 3).

Crystal defects such as vacancies, dislocations, stacking faults, and twin boundaries can provide heterogeneous nucleation sites to stimulate continuous precipitation[2]. Therefore, the spherical β-$Mg_{17}Al_{12}$ particles are often observed after aging of plastic-deformed Mg-Al alloys. A large number of TBs and small number of stacking faults and dislocations provide abundant nucleation sites resulting in a uniform distribution of precipitates in the aged UFT samples (Fig. 2a, e and g). The EDS maps (Supplementary Fig. 5) indicate that the precipitates are mainly composed of Mg and Al, together with a small amount of Zn. The segregation of Zn in β-$Mg_{17}Al_{12}$ has been reported in a Mg-9Al-3Zn alloy by Liu et al.[3]. Generally, Zn appears in the interior of β-$Mg_{17}Al_{12}$ precipitate, which is in accordance with the result in the present study.

Supplementary Tables

Supplementary Table 1 Mechanical properties of AZ80-T6 and aged UFT samples. YS and US refer to the yield strength and ultimate strength, respectively and CYS and TYS are the compression yield strength and tension yield strength, respectively. Uniform elongation is for tensile test and strain to fraction is for compressive test.

Samples	Loading type	YS (MPa)	US (MPa)	Uniform elongation/ Strain to fracture (%)	CYS/ TYS
AZ80-T6	Tension	210±4	318±10	4.9±0.6	0.63
	Compression	133±12	373±8	10.0±0.4	
Aged UFT-1	Tension	162±2	401±1	10.8±0.1	0.9
	Compression	146±1	378±4	9.1±0.1	
Aged UFT-2	Tension	275±2	435±2	10.9±1.1	0.78
	Compression	214±4	454±0	11.5±0.5	
Aged UFT-3	Tension	276±6	424±24	7.5±2.1	0.83
	Compression	228±1	452±6	9. 6±0.2	
Aged UFT-4	Tension	311±3	469±1	14.1±0.6	0.81
	Compression	252±2	466±23	9.4±0.8	

Supplementary Table 2 Results for EIS spectra fitting after immersion for 0.5 h.

sample	R_s (Ω cm^2)	Y_{dl} (Ω^{-1} cm^{-2} s^n)	n_{dl}	R_t (Ω cm^2)	Y_f (Ω^{-1} cm^{-2} s^n)	n_f	R_f (Ω cm^2)
AZ80-T6	30	0.001118	0.886	1662	0.00001132	0.942	3185
Aged UFT-4	35	0.000008942	0.937	3044	0.0006573	0.858	1573

Supplementary Table 3 Results for EIS spectra fitting after immersion for 168 h.

sample	R_s (Ω cm^2)	Y_{dl} ($Ω^{-1}$ cm^{-2} s^n)	n_{dl}	R_t (Ω cm^2)	R_L (Ω cm^{-2})	L (H cm^2)
AZ80-T6	33	0.00002284	0.897	2527	7835	3722
Aged UFT-4	34	0.00002824	0.932	5052	6225	3987

Supplementary Figures

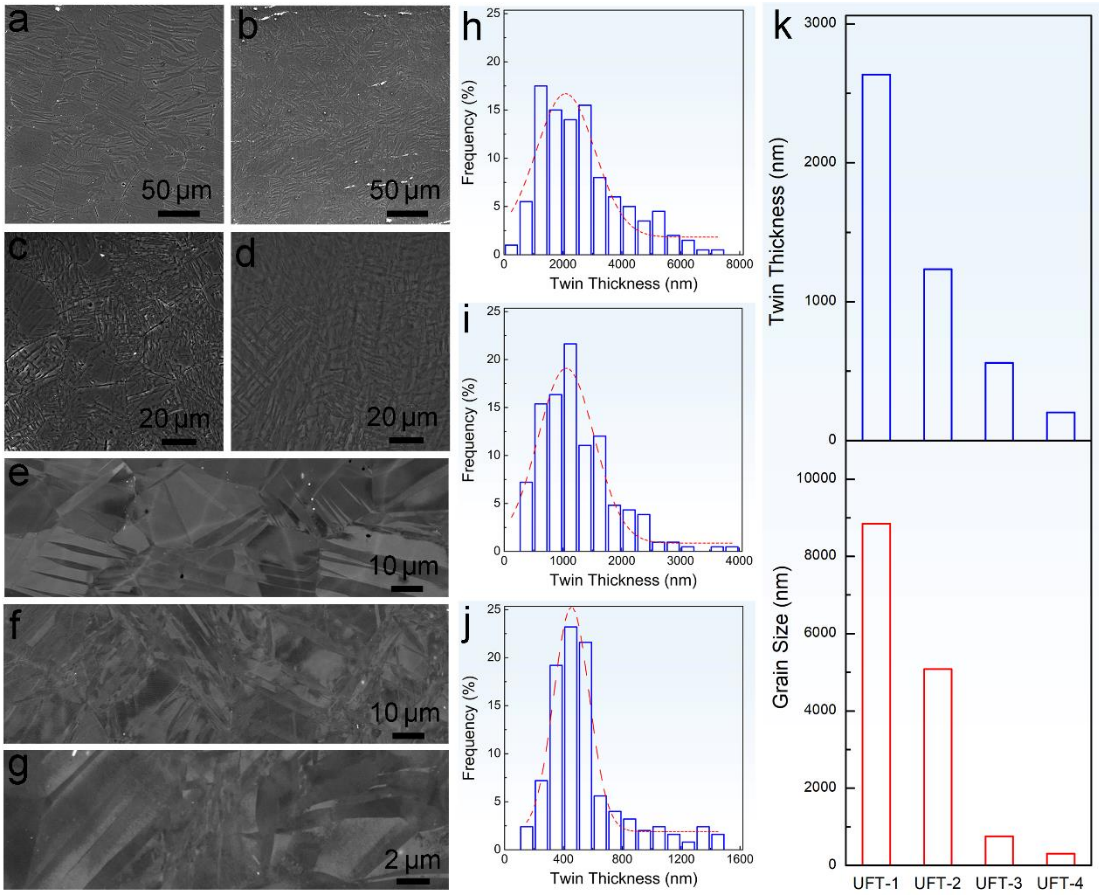

Supplementary Fig. 1 SEM images of twin microstructures: (a) UFT-1, (b) UFT-2, (c) UFT-3 and (d) UFT-4; ECC images of twin microstructures: (e) UFT-1, (f) UFT-2 and (g) UFT-3; frequency distributions of the twin lamellae thickness: (h) UFT-1, (i) UFT-2 and (j) UFT-3; (k) statistics of average twin lamellae thickness and average grain size.

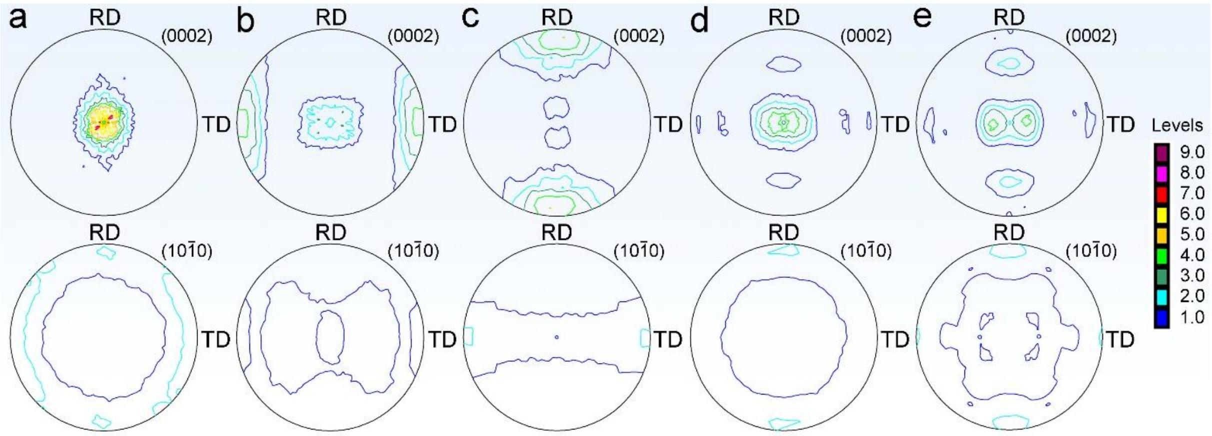

Supplementary Fig. 2 Pole figures of Mg AZ80 at different conditions determined by XRD: (a) solid solution, (b) UFT-1, (c) UFT-2, (d) UFT-3 and (e) UFT-4.

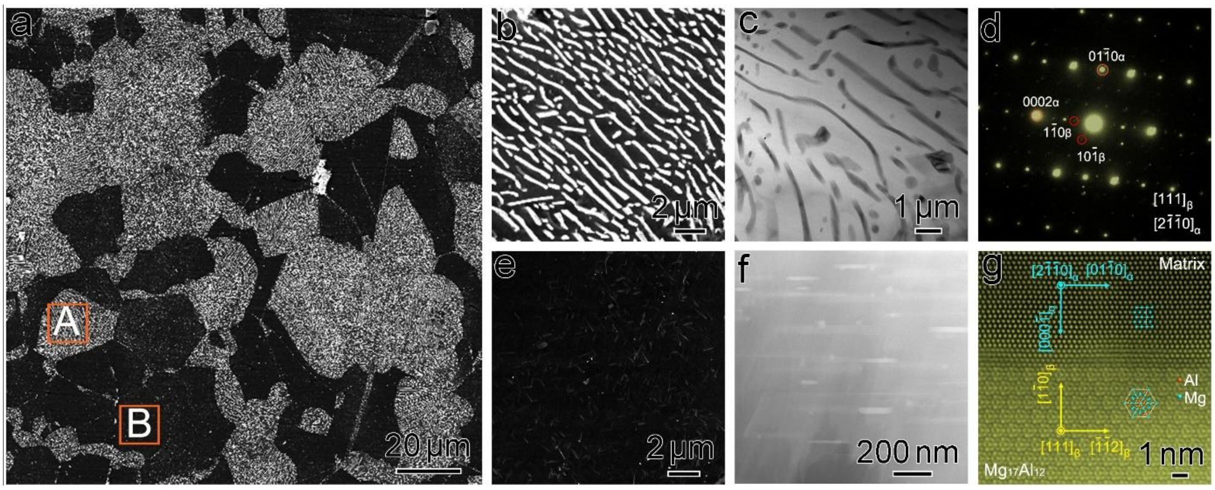

Supplementary Fig. 3 Microstructure of AZ80-T6: (a) SEM image; (b) and (e) High-magnification views of regions A and B in (a); (c) TEM image and (d) selected-area electron diffraction pattern of discontinuous β-$Mg_{17}Al_{12}$; (f) HAADF-STEM image and (g) atomic-resolution HAADF-STEM image of continuous β-$Mg_{17}Al_{12}$.

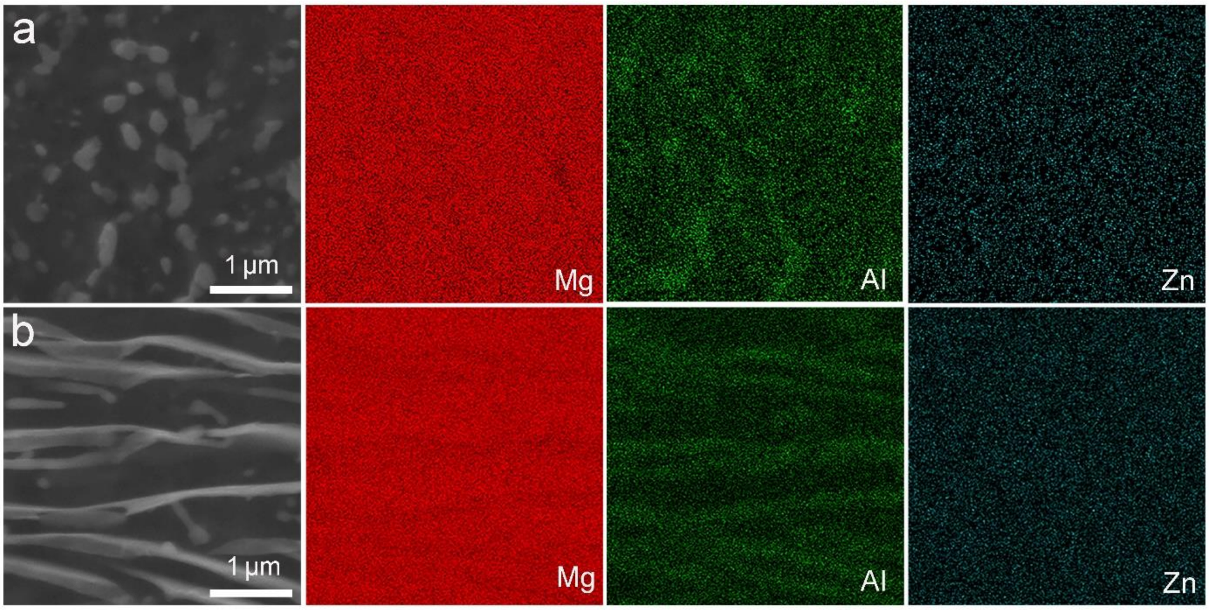

Supplementary Fig. 4 SEM images and EDS maps of β-$Mg_{17}Al_{12}$ after aging at 180 ºC for 24 h: (a) aged UFT-4 and (b) AZ80-T6.

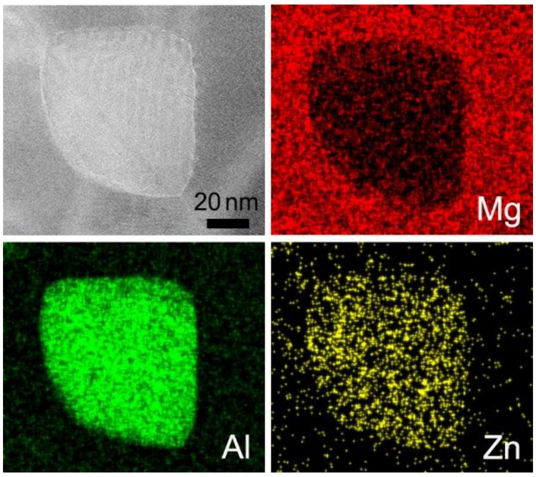

Supplementary Fig. 5 TEM image and EDS maps of β-$Mg_{17}Al_{12}$ in UFT-4 after aging at 180 ºC for 24 h.

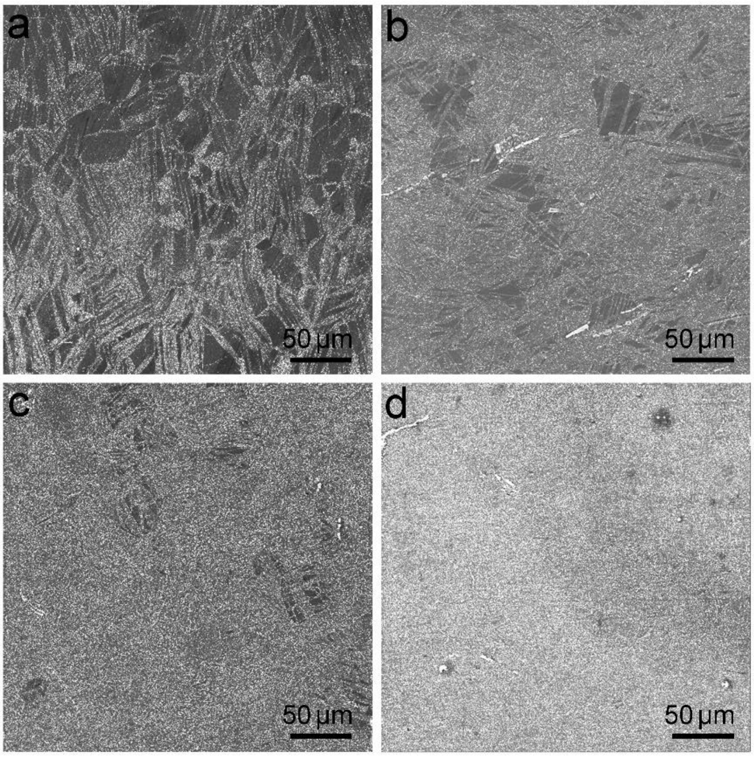

Supplementary Fig. 6 SEM images showing distributions of β-$Mg_{17}Al_{12}$ after aging at 180 ºC for 24 h: (a) UFT-1, (b) UFT-2, (c) UFT-3 and (d) UFT-4.

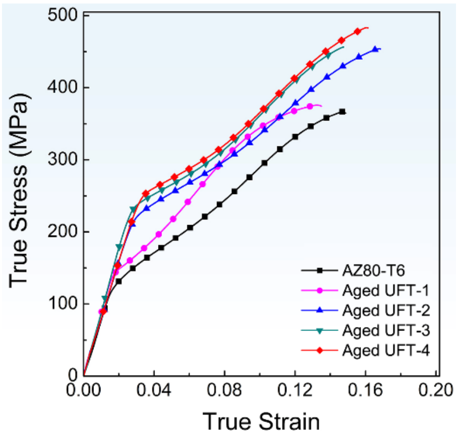

Supplementary Fig. 7 Stress-strain curves under compression along TD.

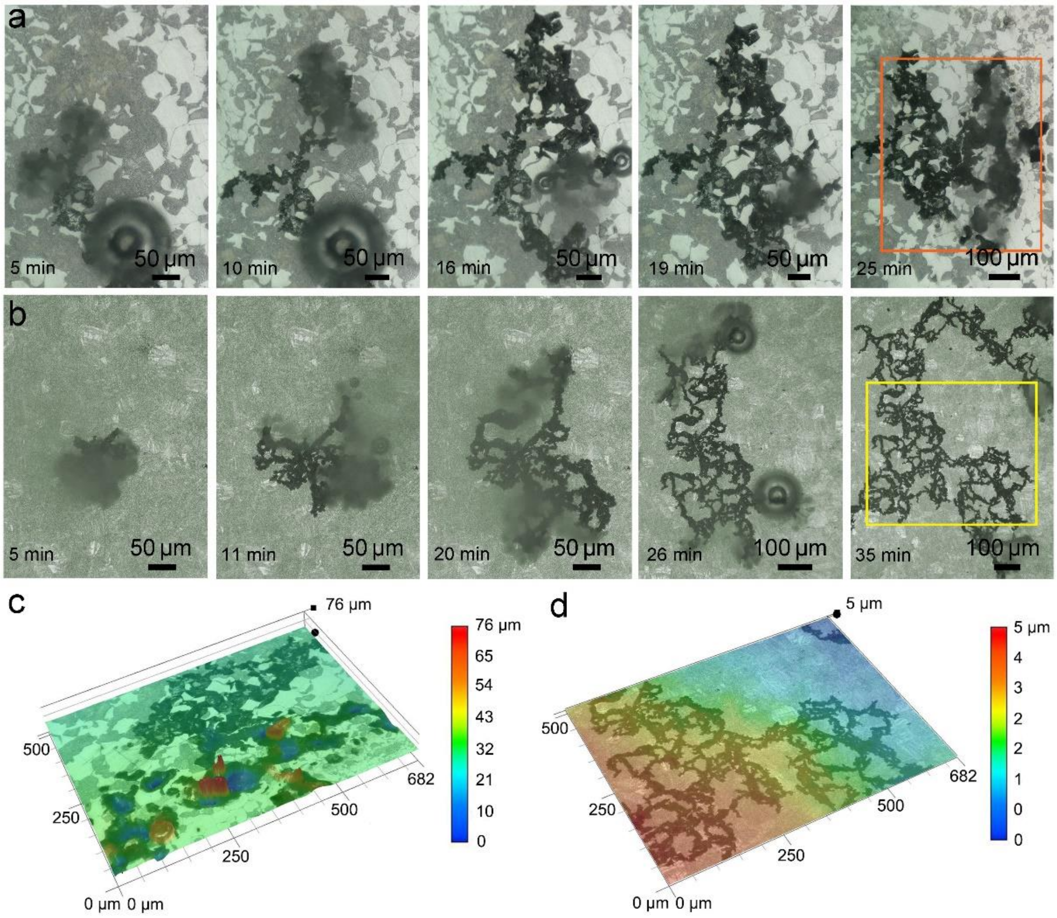

Supplementary Fig. 8 Optical images for *in-situ* observations of corrosion propagation as a function of immersion time: (a) for AZ80-T6 and (b) for aged UFT-4; three-dimensional images of the corrosion depth: (c) AZ80-T6 for 25 min and (d) aged UFT-4 for 35 min.

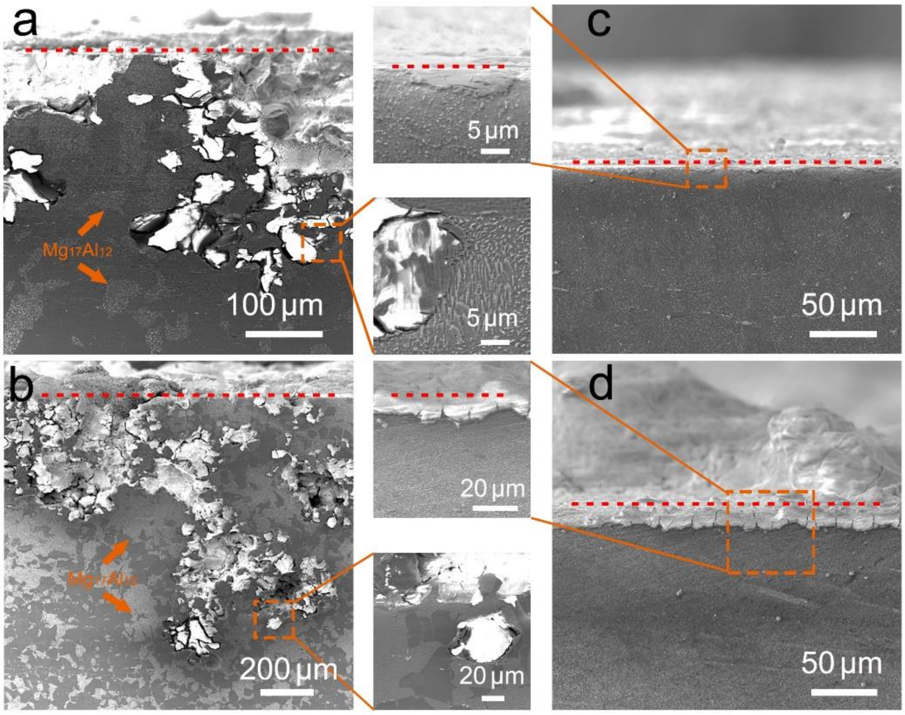

Supplementary Fig. 9 Cross-sectional views by SEM: AZ80-T6 after (a) 24 h and (b) 168 h; aged UFT-4 after (c) 24 h and (d) 168 h; high-magnification views of selected regions are denoted by orange rectangles.

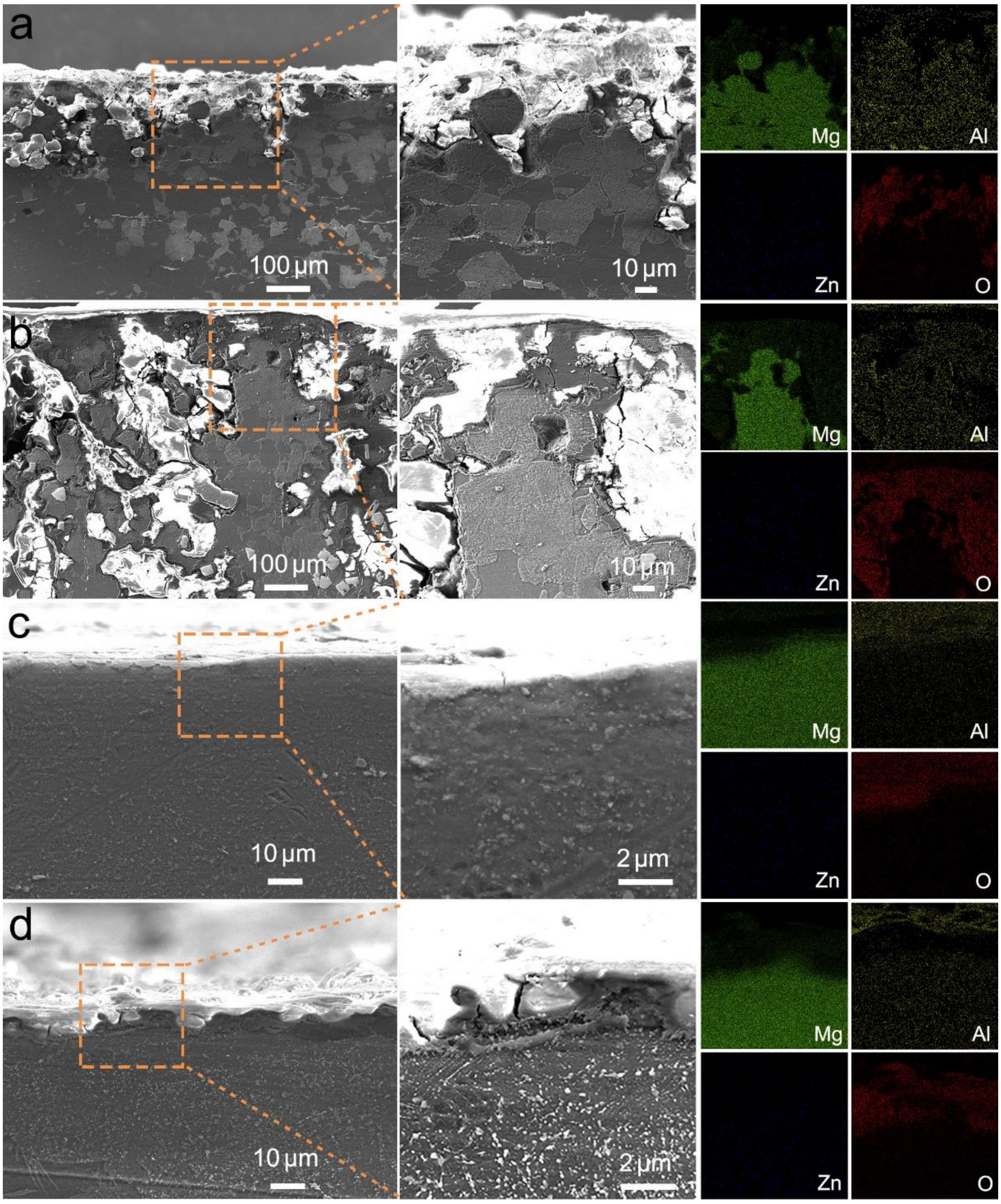

Supplementary Fig. 10 Cross-sectional views by SEM and EDS maps after exposure to 3 wt.% NaCl solution: AZ80-T6 after (a) 24 h and (b) 168 h; aged UFT-4 after (c) 24 h and (d) 168 h; high-magnification views and EDS maps of the selected regions are denoted by orange rectangles.

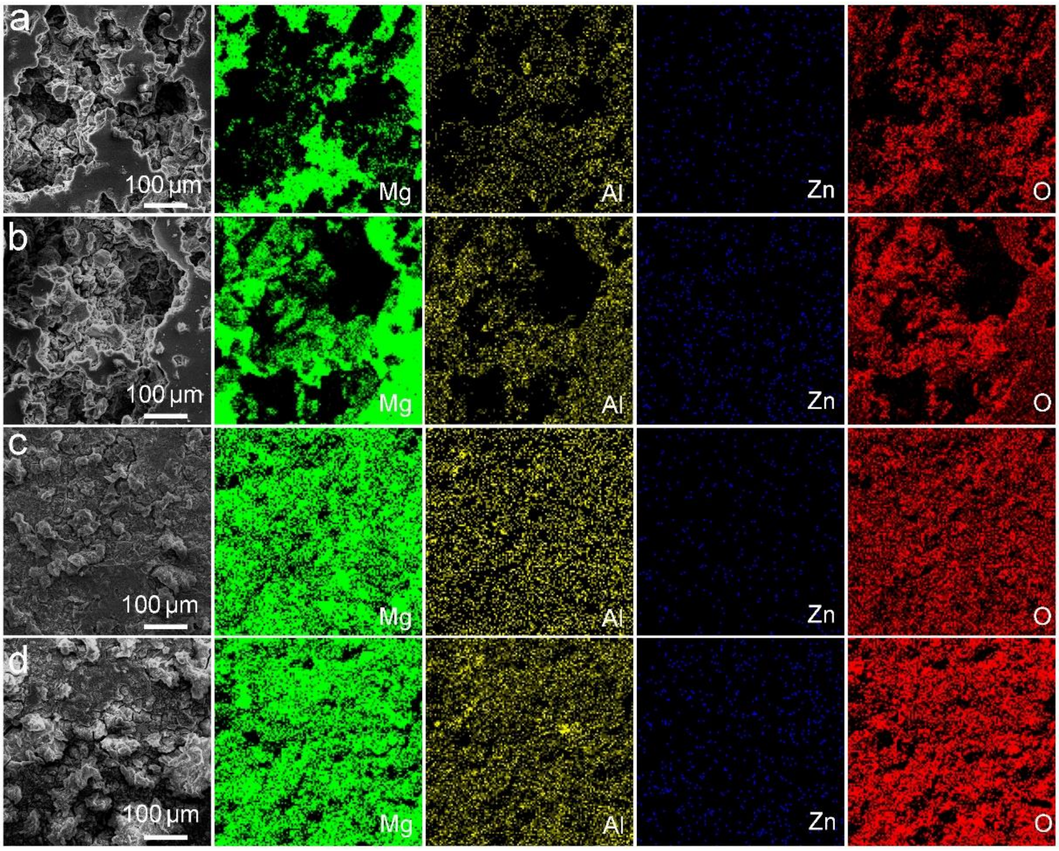

Supplementary Fig. 11 EDS maps after exposure to 3 wt.% NaCl solution: AZ80-T6 after (a) 24 h and (b) 168 h; aged UFT-4 after (c) 24 h and (d) 168 h.

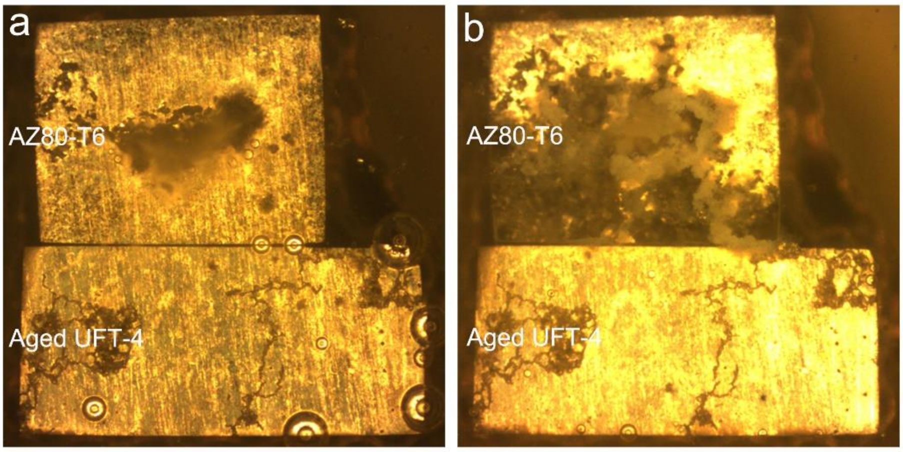

Supplementary Fig. 12 Optical images showing the corrosion morphology of the coupled AZ80-T6 and aged UFT-4 after immersion for (a) 24 h and (b) 72 h.

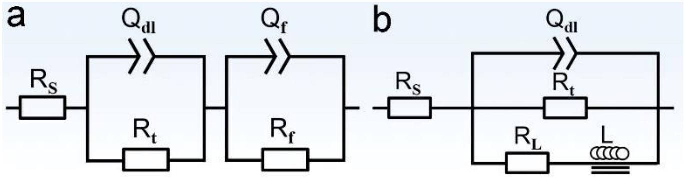

Supplementary Fig. 13 Equivalent circuit for the analysis of the EIS spectra of AZ80-T6 and aged UFT-4 after immersion in 3 wt.% NaCl solution for (a) 0.5 h and (b) 168 h. R_s – solution resistance; R_t – charge-transfer resistance; Q_{dl} – capacitance for electrical double layer; R_f – film resistance; Q_f – capacitance for corrosion product layer; R_L – inductive resistance; L – inductance.

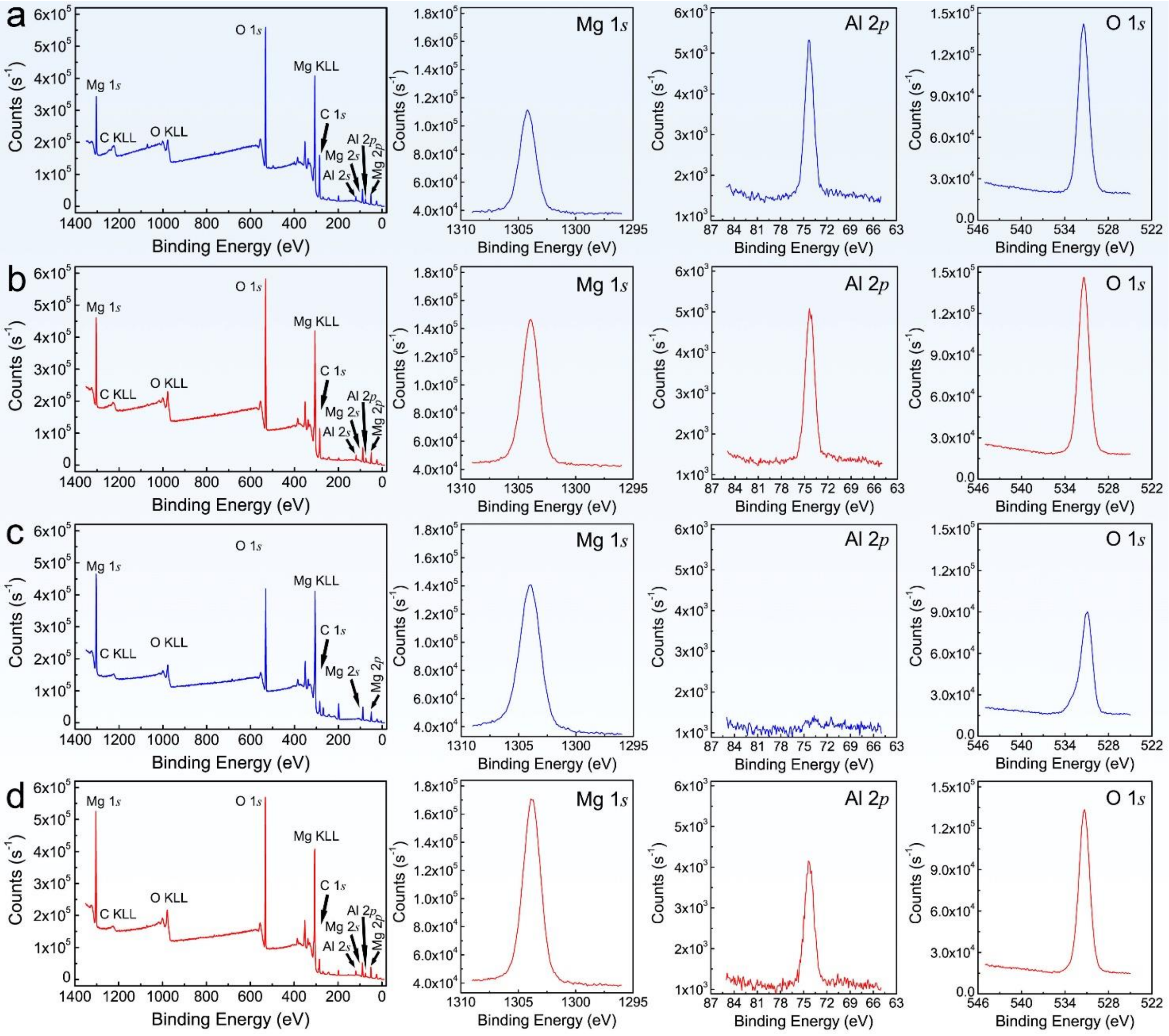

Supplementary Fig. 14 XPS spectra of the corrosion layer after immersion in 3 wt.% NaCl: (a) AZ80-T6 and (b) aged UFT-4 for 24 h; (c) AZ80-T6 and (d) aged UFT-4 for 168 h.

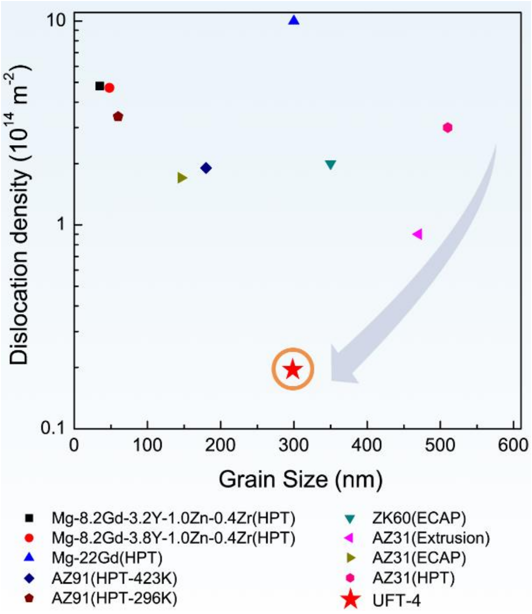

Supplementary Fig. 15 Comparison of the dislocation density between UFT-4 and Mg alloys subjected to different deformation processes (Mg-8.2Gd-3.2Y-1.0Zn-0.4Zr after HPT[4], Mg-8.2Gd-3.8Y-1.0Zn-0.4Zr after HPT[5], Mg-22Gd after HPT[6], ZK60 after ECAP[7], AZ31 after extrusion[8], AZ31 after ECAP[8], AZ91 after HPT at 423 K[9]; AZ91 after HPT at 296 K[9], AZ31 after HPT[10]).

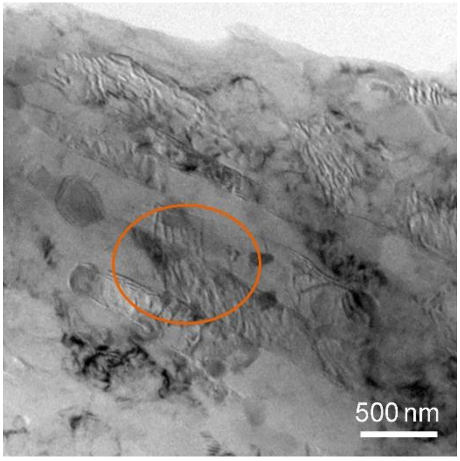

Supplementary Fig. 16 TEM image of aged UFT-4 subjected to a 3% tensile strain.

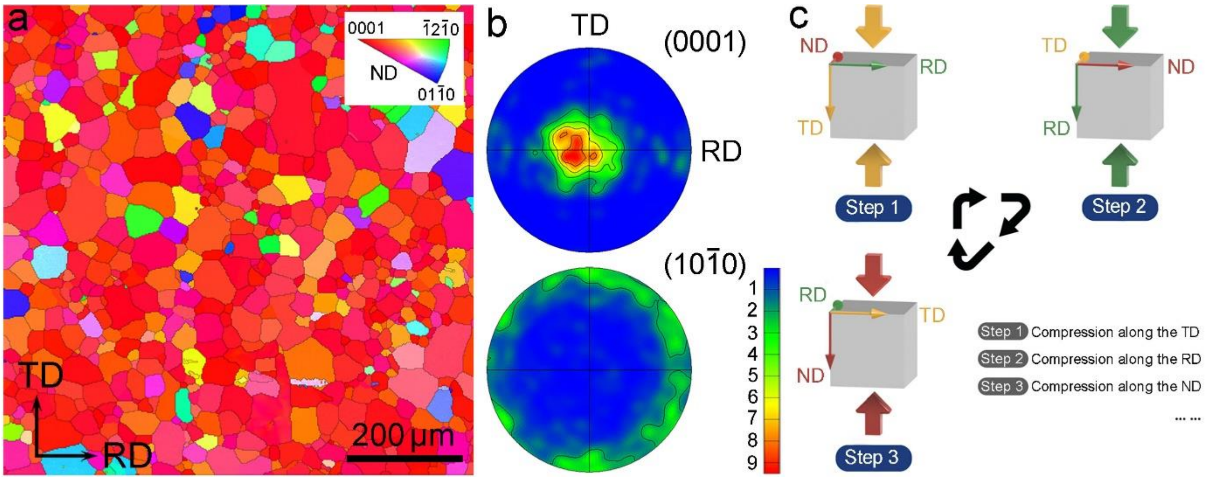

Supplementary Fig. 17 (a) Inverse pole figure map and (b) pole figures of the hot-rolled plate of Mg AZ80; (c) schematic diagram showing the multi-directional compression process.

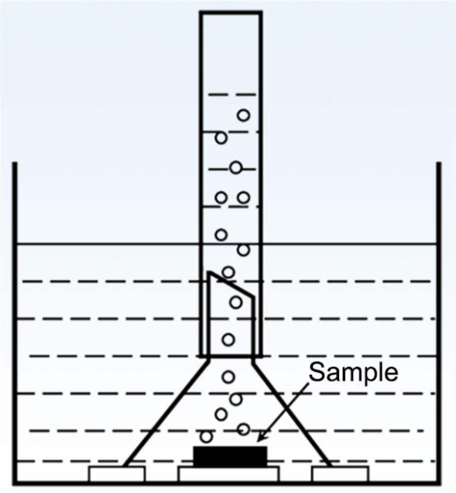

Supplementary Fig. 18 Schematic illustration of the hydrogen evolution method.

Supplementary References

[1] Braszczyńska-Malik, K.N. Discontinuous and continuous precipitation in magnesium–aluminium type alloys. *J. Alloys Compd.* **477,** 870-876 (2009).

[2] Clark, J.B. Age hardening in a Mg-9 wt.% Al alloy. *Acta Metall.* **16,** 141-152 (1968).

[3] Liu, C.Q., Chen, H.W., Wilson, N.C. & Nie, J.F. Zn segregation in interface between Mg17Al12 precipitate and Mg matrix in Mg–Al–Zn alloys. *Scr. Mater.* **163,** 91-95 (2019).

[4] Sun, W.T., et al. Achieving ultra-high hardness of nanostructured Mg-8.2Gd-3.2Y-1.0Zn-0.4Zr alloy produced by a combination of high pressure torsion and ageing treatment. *Scr. Mater.* **155,** 21-25 (2018).

[5] Sun, W.T., et al. Microstructure and mechanical properties of a nanostructured Mg-8.2Gd-3.8Y-1.0Zn-0.4Zr supersaturated solid solution prepared by high pressure torsion. *Mater. Des.* **135,** 366-376 (2017).

[6] Čížek, J., et al. Microstructure development of ultra fine grained Mg-22 wt%Gd alloy prepared by high pressure torsion. *Mater. Sci. Eng. A*. **704,** 181-191 (2017).

[7] Balogh, L., Figueiredo, R.B., Ungár, T. & Langdon, T.G. The contributions of grain size, dislocation density and twinning to the strength of a magnesium alloy processed by ECAP. *Mater. Sci. Eng. A*. **528,** 533-538 (2010).

[8] Janeček, M., Čížek, J., Gubicza, J. & Vrátná, J. Microstructure and dislocation density evolutions in MgAlZn alloy processed by severe plastic deformation. *J. Mater. Sci.* **47,** 7860-7869 (2012).

[9] Al-Zubaydi, A.S.J., et al. Evolution of microstructure in AZ91 alloy processed by high-pressure torsion. *J. Mater. Sci.* **51,** 3380-3389 (2015).

[10] Stráská, J., et al. Evolution of microstructure and hardness in AZ31 alloy processed by high pressure torsion. *Mater. Sci. Eng. A*. **625,** 98-106 (2015).
